# 2D Material Science: Defect Engineering by Particle Irradiation

**DOI:** 10.3390/ma11101885

**Published:** 2018-10-02

**Authors:** Marika Schleberger, Jani Kotakoski

**Affiliations:** 1 Fakultät für Physik and Cenide, Universität Duisburg-Essen, Lotharstraße 1, 47057 Duisburg, Germany; 2 Fakultät für Physik, Universität Wien, Boltzmanngasse 5, 1090 Wien, Austria; jani.kotakoski@univie.ac.at

**Keywords:** 2D materials, ion irradiation, electron irradiation, graphene, transition metal dichalcogenides, boron nitride, defect engineering

## Abstract

Two-dimensional (2D) materials are at the heart of many novel devices due to their unique and often superior properties. For simplicity, 2D materials are often assumed to exist in their text-book form, i.e., as an ideal solid with no imperfections. However, defects are ubiquitous in macroscopic samples and play an important – if not imperative – role for the performance of any device. Thus, many independent studies have targeted the artificial introduction of defects into 2D materials by particle irradiation. In our view it would be beneficial to develop general defect engineering strategies for 2D materials based on a thorough understanding of the defect creation mechanisms, which may significantly vary from the ones relevant for 3D materials. This paper reviews the state-of-the-art in defect engineering of 2D materials by electron and ion irradiation with a clear focus on defect creation on the atomic scale and by individual impacts. Whenever possible we compile reported experimental data alongside corresponding theoretical studies. We show that, on the one hand, defect engineering by particle irradiation covers a wide range of defect types that can be fabricated with great precision in the most commonly investigated 2D materials. On the other hand, gaining a complete understanding still remains a challenge, that can be met by combining advanced theoretical methods and improved experimental set-ups, both of which only now begin to emerge. In conjunction with novel 2D materials, this challenge promises attractive future opportunities for researchers in this field.

## 1. Introduction

Modern material science aims at improving physical and chemical properties of solids to advance their use in applications. In the original state of a material these properties may be either inadequate, insufficient, or even completely absent. By combining physics, chemistry and engineering in clever ways, scientists have succeeded in tuning the original properties of many a material. Arguably, the best-know example is the semiconductor industry where an impressive control of the electronic properties in combination with band-gap engineering has made innovative applications such as efficient light emitting diodes or quantum well lasers possible [1]. Common strategies for tuning material properties employ outer parameters such as temperature, pressure, and strain, but also intrinsic quantities, e.g., the density and type of defects and dopants. However, these strategies have all been traditionally developed and optimized for bulk materials. For two-dimensional materials, consisting of only from one up to three atomic layers, strategies affecting the intrinsic quantities have to be adapted and sometimes even abandoned as they are no longer effective. For example, doping silicon by ion irradiation is routinely achieved with a high level of precision [2] while doping of graphene by ion irradiation still poses a challenge [3,4,5]. However, 2D materials offer also completely new possibilities. For example, band engineering in 3D is constrained by the laws of epitaxial crystal growth while 2D materials can be simply stacked onto each other [6].

Since the discovery of graphene by Geim and Novoselov some ten years ago [7] there have been countless suggestions for novel applications based on 2D materials. Many of them are based on the – sometimes implicit – assumption that the 2D material is in its perfect, intrinsic state while for others imperfections represent the key feature, e.g., size selected pores for desalination [8,9], energy harvesting [10,11], and DNA sequencing [12,13,14,15], or edge states for spin currents [16] and catalytic activity [17,18]. As a consequence, 2D material science needs to address both aspects, to investigate and account for ubiquitous defects and foreign atoms, and to develop efficient strategies to control material properties by introducing imperfections in a reliable way [19,20,21]. In 2D materials the tuning of intrinsic material properties inevitably proceeds via modifications at the atomic scale. Thus, 2D material science deals with defect engineering in the broadest sense, i.e., enabling the controlled removal, addition or manipulation of atoms and as such represents the key to unlock the full potential for applications. This paper reviews what has been achieved in this respect by means of particle irradiation, one of the most important tools for material modifications at the nanoscale. Complementary to the paper by Li et al. which gave an excellent review on ion beam modifications of 2D materials [22] we include electrons as an important tool in particular in combination with high resolution electron microscopy and we put a special emphasis on high energy ions which may be used to create unique types of defects.

We begin this paper by reviewing briefly the different physical mechanisms of particle-solid interaction, from which the 2D material scientist may choose by selecting a certain type of beam. We will then give an overview over what has been achieved for various 2D materials with the respective beam types and discuss the current state-of-the-art of defect engineering by particle irradiation. We close our paper with a synoptic view of open problems.

## 2. Particle Beams Interacting with Solids

### 2.1. Ion Beams

The interaction of ions with solid matter has been researched for more than a century now. Since the discovery of Rutherford and co-workers that alpha particles shot at a gold foil undergo scattering events with – at that time – unexpectedly large scattering angles, it has been established that the collision of an ion with another atom can be understood in terms of scattering in a (screened) Coulomb potential

(1) $$V\left(r\right)=\frac{1}{4\pi \epsilon_{0}}\frac{Z_{1}Z_{2}e^{2}}{r}\cdot \phi \left(r\right),$$

with *Z* being the atomic number of the projectile (subscript 1) and the target (subscript 2), *e* elementary charge, and $\epsilon_{0}$ the dielectric constant. Screening effects due to the electron cloud can be accounted for by an appropriate screening function ϕ(r), see e.g., [23]. In such a scattering event kinetic energy of the projectile is transferred to target atoms via momentum transfer and may lead to the displacement of one or more target atoms. The maximum energy transfer from a projectile with mass $m_{1}$ and velocity $v_{1}$ to an atom with mass $m_{2}$ at rest ($v_{2}=0$) is given by

(2) $$E_{T},\max=\frac{4m_{1}m_{2}}{{\left(m_{1}+m_{2}\right)}^{2}}\frac{m_{1}v_{1}^{2}}{2},$$

and the differential cross section for the energy $E_{T}$ to be transferred in a scattering event is given by Thomson’s formula:

(3) $$\sigma \left(E_{T}\right)=\frac{2\pi {\left(Z_{1}Z_{2}e^{2}\right)}^{2}}{m_{2}v_{1}^{2}}\frac{1}{E_{T}^{2}}.$$

In case of an extended lattice and sufficient kinetic energy of the projectile, more complex processes such as the evolution of a collision cascade [24] may evolve. All these processes are elastic in nature in the sense that the kinetic energy of the projectile is transformed into kinetic energy of the recoiling collision partners. These scattering processes are commonly referred to by the term *nuclear stopping*. For easier comparison the amount of energy that is deposited in the material is given in terms of a friction force, i.e., energy per unit track length

(4) $$S_{n}=-dE_{n}/dx.$$

Due to the fact that 2D materials are only one to three atomic layers thick, the collision cascade in their case is mostly absent, which influences e.g., sputtering rates but also doping efficiencies. Also, typical defect types obtained in 2D materials by individual impacts of low energy ions are point-like defects, as will be discussed in more detail below.

For very fast (There is no exact definition of fast, but often a specific energy larger than 0.1 MeV/amu is meant.) and heavy (Again, the definition remains diffuse, but heavy often means anything heavier than carbon.) ions however, the situation changes considerably. This is due to the swift movement of the projectiles that enables processes involving charge exchange and ionization and/or electronic excitation of target atoms and the projectile. The subsequent relaxation processes often leave the target material in a structurally and/or electronically changed state, providing a basis for defect engineering. This became evident to material scientists early on, for example, from inspecting the helmets of Apollo astronauts [25], and is today exploited, e.g., in cancer therapy [26]. These processes are inelastic in nature and are summarized under the term *electronic stopping*, where the energy deposition is again given in terms of energy per unit track length

(5) $$S_{e}=-dE_{e}/dx.$$

**Figure 1:**
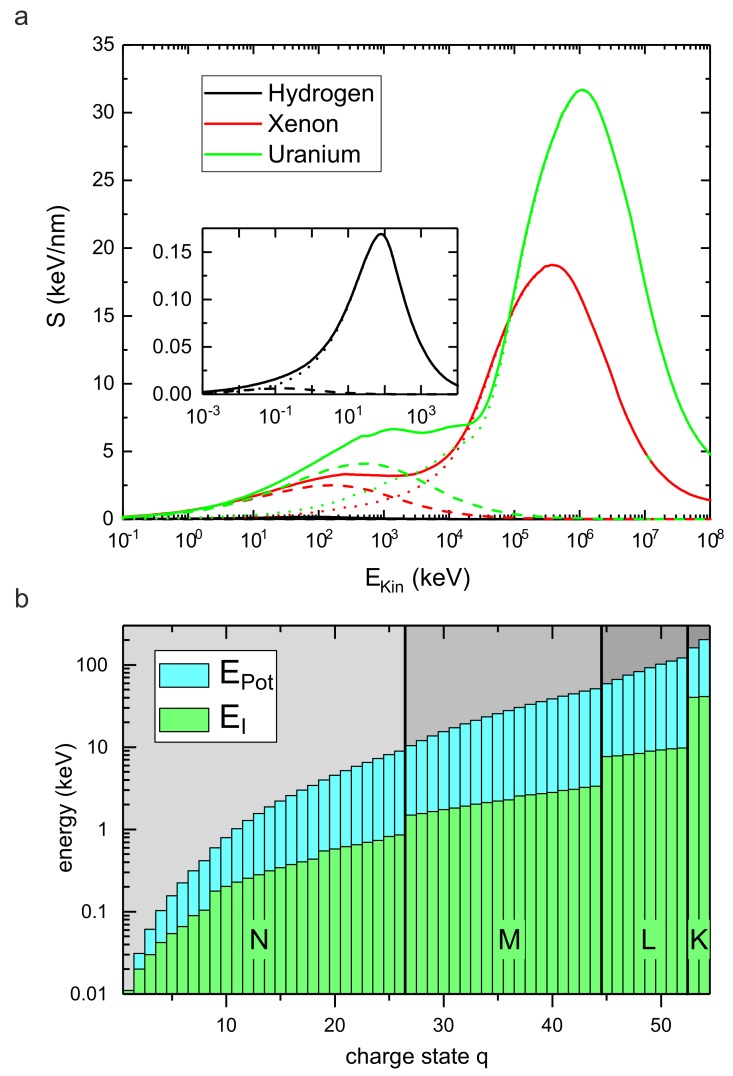
Energy deposition by swift heavy and highly charged ions. (**a**) Total stopping power *S* (continuous lines) of various ion species (H, Xe, U) in graphite as a function of the kinetic energy of the projectile calculated with SRIM. The contribution of nuclear stopping $S_{n}$ is plotted with dashed lines, of electronic stopping $S_{e}$ with dotted lines. The data for protons is shown in the inset. (**b**) Potential energy $E_{pot}$ of a Xe ion as a function of its charge state *q* (blue bars). The green bars depict the ionization energy $E_{I}$ of the last electron. Jumps in the ionization energy occur after an electron shell has been completely emptied.

The detailed theoretical description was developed by Bohr, Bethe and Bloch [27,28,29] taking also higher velocities into account, and later-on refined by Lindhard [30] and others. Up to projectile velocities of $v=v_{Bohr}Z_{1}^{2/3}$ the equilibrium charge state changes as $Q_{eq}=Z_{1}^{1/3}\cdot v/v_{Bohr}$ until the projectile becomes fully ionized. In this energy regime the electronic stopping can be understood as a friction acting on the projectile and $S_{e}$ thus scales roughly with its velocity, i.e., with $\sqrt{E_{Kin}}$. At even higher energies $S_{e}$ is inversely proportional to $E_{Kin}$. This dependence of $S_{e}\left(E_{Kin}\right)$ can be seen in Figure 1a.

The deposited energy may dissipate in the target material in various ways, some of which are associated with material changes. As physical mechanisms for example electron-phonon-coupling and thermal spikes [31,32,33], non-thermal melting [34], Coulomb explosion [35], and exciton self-trapping [36] have been suggested. As a consequence, the occurrence of permanent material changes does strongly depend on the material properties. From the research on bulk materials, the following rule-of-thumb has been established: insulators are the easiest to modify, followed by semiconductors and metals, the latter being to a large extent insensitive to ionizing radiation. This radiation hardness is due to the quick spreading of the deposited energy of the excitation, which leads to insufficient energy densities over long times.

For practical calculations of the energy deposition by ion irradiation, a software package SRIM (The Stopping and Range of Ions in Matter) was developed by Ziegler et al. [37]. Figure 1a shows the two contributions to the total stopping $S=S_{n}+S_{e}$ as a function of the projectile’s kinetic energy for the case of hydrogen, xenon, and uranium, respectively, penetrating graphite (density $\rho =2.26\;g/{\mathrm{cm}}^{3}$) as calculated by SRIM. The dependence on $E_{\mathrm{kin}}$ and on the mass of the projectile is clearly seen.

**Figure 2:**
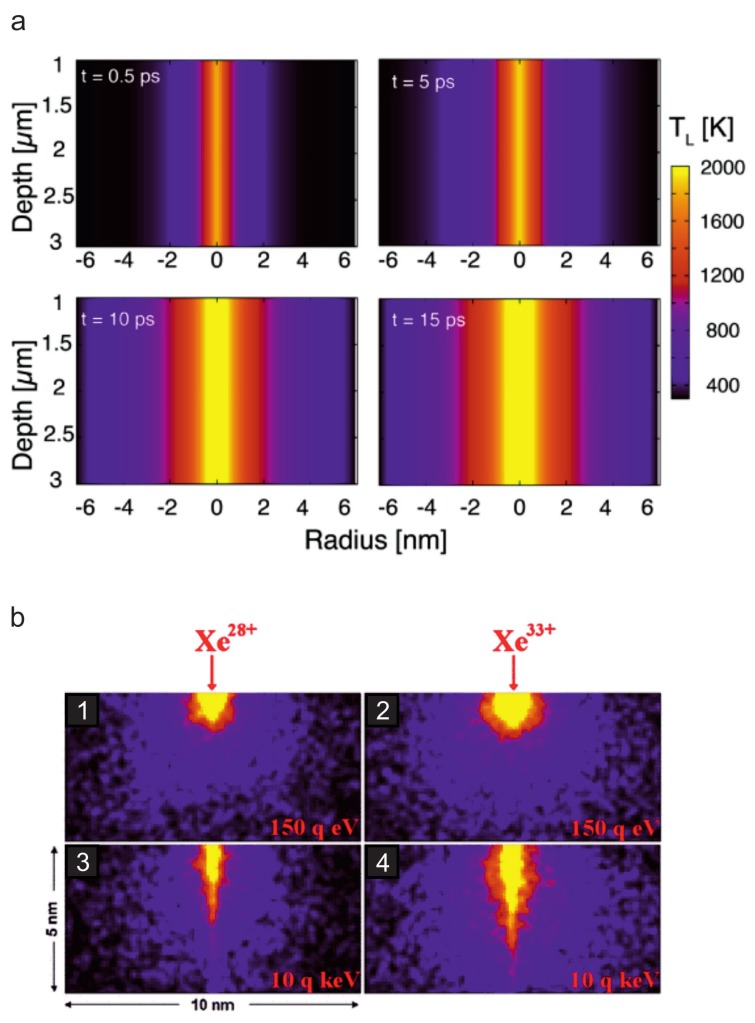
Evolution of the lattice temperatures due to ion impact calculated with the two temperature model showing the different depths of energy deposition of SHI and HCI. (**a**) Lattice temperatures for an SHI (11.4 MeV/amu Ca with dE/dx=2.6 keV/nm) traversing crystalline SiO${}_{2}$. From Ref. [43]. © IOP Publishing. Reproduced with permission. All rights reserved. (**b**) Lattice temperatures for an HCI (Xe${}^{28+}$ and Xe${}^{33+}$) in CaF${}_{2}$ for two different kinetic energies (150×q eV and 10×q keV). Reprinted with permission from Ref. [44], Copyright (2011) by the American Physical Society. Note the different *z*-scales for the two different ion types.

At low kinetic energies nuclear stopping dominates, while at higher kinetic energies electronic stopping is the dominant process. The typical electronic stopping of swift heavy ions (SHI) in most solids is on the order of a few keV per nm. The maximum energy loss (e.g., S≃32 keV/nm in the case of uranium, see Figure 1a) is generated at $v_{Bohr}Z_{1}^{2/3}$, where the excitation of the electron gas is most effective and the interaction time is still sufficiently long. In general, SHI are not easily stopped. The mean range $R_{m}$ of a projectile with initial energy $E_{0}$ is given by

(6) $$R_{m}=\int_{E_{0}}^{0}\frac{1}{S(E)}dE,$$

thus their straight trajectories typically extend deep into the material and can easily reach several tens or even hundreds of microns, see Figure 2a. As a consequence, the interaction volume with a 2D material compared to the total interaction volume is extremely small under perpendicular incidence. For defect engineering of 2D materials it can therefore be advantageous to tilt the beam with respect to the surface so that the interaction length is significantly enlarged. For example, in the case of graphene an interaction length of several 100 nm can be achieved at oblique angles of incidence Θ, in contrast to 3 Å at $\Theta =90^{\circ }$ (as discussed in detail in subsection 3.3). However, in the case of supported samples the interaction is never limited to the 2D material itself, and will instead always affect the substrate as well which will in turn affect and sometimes obscure the defect creation in the 2D material.

Irradiation by SHI is unique in the sense that the corresponding defect engineering mechanism relies on electronic excitations and usually requires large-scale infrastructure as the ion beams with necessary kinetic energies are obtained from van-de-Graaff and linear accelerators or cyclotrons. However, electronic excitations cannot only be triggered by fast projectiles but also by sufficiently charged projectiles. With the development of modern ion sources a new type of ion beam became accessible for material science in the 1980’s, when highly charged ions (HCI) could be extracted reliably from compact electron beam ion traps [38] replacing the electron cyclotron resonance (ECR) ion sources. As a consequence the interaction of HCI with solids was intensively studied, although at that time mostly from the view point of the projectile [39]. In a way, these ions represent a mixture of the two ion types discussed above as they typically have a kinetic energy in the nuclear stopping regime but at the same time excite the electronic system due to the deposition of their high potential energy

(7) $$E_{pot}\left(q\right)=\sum_{i=1}^{q}E_{I}^{i},$$

which is the sum of the ionization energies $E_{I}$ of the detached electrons and thus depends on the charge state *q*, see Figure 1b. The achievable energy densities are comparable to or may even exceed those achieved by SHI, because in the case of HCI the energy is deposited into a very small volume near the surface (see Figure 2b). This is not only due to the lower kinetic energy of HCI with respect to SHI, but also related to its specific interaction with the target material: Upon approach, an image charge builds up in the target material. For a metal, this image charge in turn increases the kinetic energy of the projectile by $\Delta E_{kin}^{image}\left(W,q\right)≃\frac{W}{3\sqrt{2}}q^{3/2}$, where *W* is the work function of the target material. As soon as a critical distance $r_{crit}=\sqrt{2q}/W$ is overcome [40], charge transfer sets in releasing the potential energy stored in the projectile. The HCI is neutralized by the captured electrons which are occupying predominantly high-energy Rydberg states. The relaxation of this *hollow atom* proceeds via a number of processes like Auger and quasi-resonant neutralization, Auger and resonant ionization, and radiative de-excitation.

In the case of graphene it has recently been shown by carefully analyzing the exit charge state in transmission experiments that interatomic Coulombic decay is the main mechanism for energy deposition, leading to a release of low energy electrons from graphene [41,42]. Similar data on other 2D materials does not yet exist. In general, the subsequent energy transfer processes within the target material relevant for defect engineering are not yet well understood and a satisfying theoretical model with predictive power with respect to number and type of defects for a given HCI impact is still lacking. Nevertheless, as potential and kinetic energy may be varied independently and the potential energy deposition is limited to the surface, these projectiles seem to be in particular well suited for defect engineering of 2D materials. Systematic studies on this topic, however, are only about to emerge.

### 2.2. Electron Beams

Despite the much lower mass of electrons as compared to ions, electron impacts can also be used for manipulation of materials. This is typically done in the context of transmission electron microscopy (TEM), where the electron energies are in the range of 40–300 keV. Due to the limited field of view, manipulation of materials with TEM is often restricted to relatively small areas (some hundreds of nm${}^{2}$), but it can be carried out with simultaneous atomic-resolution observation. Modern scanning transmission electron microscopy (STEM) devices further allow focusing the electron beam down to ∼1 Å, providing the opportunity to target individual atoms in 2D materials.

Similar to ion irradiation, electron irradiation-induced structural changes in TEM are divided into ionization damage (due to inelastic scattering of impinging electrons) and knock-on damage (elastic scattering resulting in momentum transfer from an electron to a nucleus in the sample). Which of the two mechanisms is important in a particular experiment depends on the electron energy and the target material. Although they are unwanted when transmission electron microscopy is used purely for imaging samples, they can also be utilized for defect-engineering, provided that their roles are properly understood and can be experimentally controlled.

Out of the two mechanisms, knock-on damage is simpler to understand. In the case of conducting samples, knock-on damage dominates over ionization due to the quick replenishment of electrons (on the time scale of 1 fs [45], short enough to prevent significant atomic motion) when they are removed by the electron beam. Knock-on process can be described as Coulomb scattering of relativistic electrons by atomic nuclei using the conditionally convergent infinite series developed by Mott, for which McKinley and Feshbach developed a simple approximation in 1948 [46]. This approximation (and other similar ones) have been used effectively over the years to estimate electron beam damage in different materials, including carbon nanostructures [47], often assuming that the displacement threshold ($T_{d}$, minimum kinetic energy required to remove an atom from the lattice) can be approximated to be isotropic and that the energy transfer from the electron beam can be described using the largest possible energy transferred from an electron with energy $E_{e}$ to a target atom with mass *M* at rest ($v_{z}=0$, where *z* is the direction of the electron beam)

(8) $$E{\left(v_{z}=0,E_{e}\right)}_{\max}=2E_{e}\left(E_{e}+2m_{e}c^{2}\right)/Mc^{2},$$

where $m_{e}$ is the mass of an electron and *c* the speed of light. However, it has been known since decades [48], that this is in fact a significant simplification and the velocity distribution of the target atoms must be taken into account at electron energies close to the damage threshold. Taking into account that the kinetic energy of the electron ($E_{e}$) is orders of magnitude larger than those of the nucleus before ($E_{n}$) or after (*E*) the collision ($E_{e}+E_{n}-E\approx E_{e}$), the maximum transferred kinetic energy from the electron to an atom with velocity $v_{z}$ becomes

(9) $$E{\left(v_{z},E_{e}\right)}_{\max}=\frac{{\left(2\sqrt{E(E+2mc^{2})}+Mv_{z}c\right)}^{2}}{2Mc^{2}}.$$

It has been shown recently through atomic-resolution knock-on experiments with graphene using high resolution TEM (HRTEM) and scanning TEM (STEM) [49,50,51] that a simple modification to the formula by McKinley and Feshbach taking into account the lattice vibrations leads to a nearly perfect agreement between measured and calculated cross section values for electron energies between 80 and 100 keV. The agreement was obtained using a mean square velocity calculated for graphene using its phonon density of states from density functional theory (DFT) [51]. The cross section becomes

(10) $$\sigma \left(T,E_{e}\right)=\int_{E_{\max}\left(v_{z},E_{e}\right)\geq T_{d}}P\left(v_{z},T\right)\sigma \left(E_{\max}\left(v_{z},E_{e}\right)\right)dv,$$

where *T* is the temperature, $E_{e}$ electron energy, $E_{\max}\left(v_{z},E_{e}\right)$ maximum energy transferred from the electron to a target atom with velocity $v_{z}$, $T_{d}$ displacement threshold of the material, and $P(v_{z},T)$ the velocity distribution of the atoms in the material in the direction of the electron beam. The $\sigma (E_{\max}\left(v_{z},E_{e}\right))$ is the original cross section from the McKinley-Feshbach approximation, modified to include the effect of C atom velocity $v_{z}$ on the transferred energy. The only unknown parameter in the theoretical model is $T_{d}$ (21.14 eV for graphene obtained through a fit; ca. 21.3 eV as predicted by DFT-based dynamical simulations [51]). The resulting excellent agreement between the theory and the experiments confirms that knock-on damage indeed dominates for conducting samples and that it can be adequately described with the existing model.

Cross sections for ionization events are several orders of magnitude higher than those for the knock-on damage, with an increasing trend towards lower electron energies due to increasing interaction between the impinging electrons and electrons in the sample. The total cross section of inelastic scattering $\sigma_{i}$ can be measured from the electron energy loss, since [45]

(11) $$\sigma_{i}=\int \left(d\sigma_{i}/dE\right)dE={\left(In_{a}t\right)}^{-1}\int \left(dJ/dE\right)dE,$$

where *I* is the electron beam intensity (current), $n_{a}$ the number of atoms per volume and J(E) the energy-loss spectrum. However, since this cross section includes also inelastic collision events that do not result in bond breaking, the actual expected damage will have a cross section lower than this. As mentioned, ionization appears irrelevant in the case of graphene, but plays a significant role for insulating and semiconducting materials, as is clear from early results for two-dimensional hexagonal boron nitride (hBN) [52] and molybdenum disulfide MoS${}_{2}$ [53]. For example, for MoS${}_{2}$, the knock-on damage cross section is estimated based on DFT simulations to be in the order of 1 barn, whereas the experimental estimate for total damage cross section (including the contribution from ionization) is approximately twice as high [53]. However, there is currently not enough data that would allow an accurate comparison between the two estimates (on experimental side, better statistics obtained at different electron energies are needed, and also the theoretical description should be improved, similar to what was done for graphene in Ref. [51]).

Ionization effects can be to some extent (by a factor of 3–10 [45]) mitigated through cooling of the sample with liquid nitrogen or helium, which provides sufficient time for the electronic system to recover before the structure decomposes. Another possibility, available for two-dimensional materials [54], is to place the non-conducting material (e.g., MoS${}_{2}$) onto a good conductor (e.g., graphene), which can replenish the electrons at a quick enough pace to prevent damage.

## 3. Defect Engineering by Particle Irradiation: State of the Art

### 3.1. Electrons

**Figure 3:**
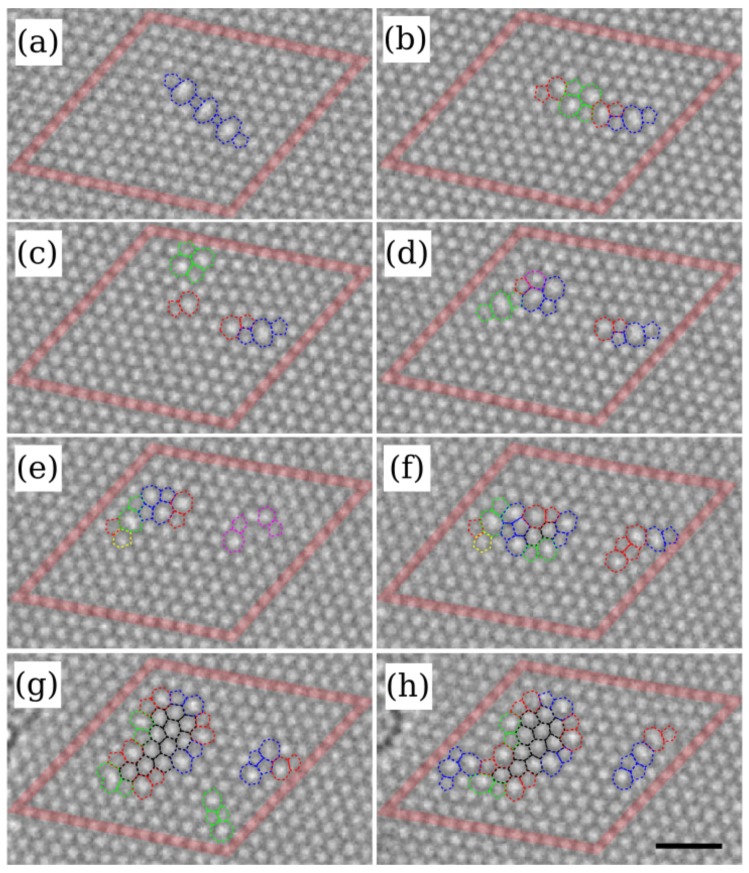
Formation and growth of vacancy-type defects with rotated-hexagon kernels in multivacancy structures. The structural changes were obtained under 100 keV electron irradiation. The images are eight subsequent frames recorded during the experiment. The initial configuration in (**a**) consists of three divacancies in the armchair orientation, in the final configuration (**h**) 24 atoms are missing. Scale bar is 1 nm. Reprinted with permission from Ref. [55], Copyright (2011) by the American Physical Society.

The simplest defect in any material is the single vacancy, in which one lattice site of a crystal is vacant. Due to the typical fluxes in TEM devices (1 $e^{-}$/nm${}^{2}$/ns) ensuring just one electron in the microscope column at any given time (electrons traverse the column in about 10 ns), each electron impact on an atom in the sample can be treated as a separate event. The low mass of the electron further limits the kinetic energy transfer to only a few eV (up to 20 eV for light elements such as carbon at 100 kV). As a result, for a pristine crystal, knock-on damage typically leads to the creation of a single vacancy. However, after the first vacancy has been created, more complicated defects can follow. Evolution of vacancy-type defects in graphene under electron irradiation are shown in Figure 3.

From the damage perspective, it is often crucial that the removal of atoms is prevented. For knock-on damage, the only feasible way to do this is to lower the electron energy (i.e., used acceleration voltage) to a value low enough to make the displacement process so unlikely that high signal-to-noise images of the sample can be obtained without the loss of atoms. This has resulted in the recent push towards lower acceleration voltages [56], enabled by advances in correcting electron optical aberrations. Due to this development, many microscopes are now operated at 60 or 80 kV (or below), which reduces the knock-on cross section to practically zero for graphene.

**Figure 4:**
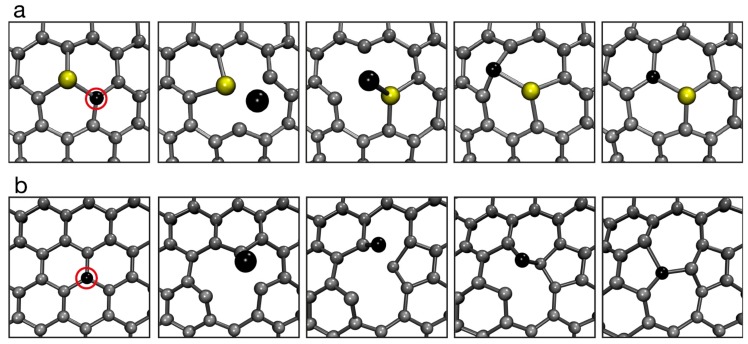
Ab initio molecular dynamics simulations of atom-number-conserving transformations in graphene. (**a**) Migration step of a Si impurity atom caused by an electron impact on its neighboring carbon atom (marked with a red circle). (**b**) Stone-Wales transformation caused by an electron impact on one of the carbon atoms (marked with a red circle). From Ref. [64].

Experiments at these low voltages have revealed that the simple removal of an atom is not the only structure-altering mechanism enabled by the elastic scattering, even for the one-atom-thick graphene. It turns out that momentum transfer below the knock-on threshold is in some cases sufficient to momentarily displace the atom away from its lattice position, during which time the other atoms can rearrange so that the returning displaced atom will end up in a different atomic configuration than initially. For defect-free graphene, this process can lead to a bond rotation (often called *Stone-Wales transformation*) [55,57], whereas in already defected areas it can drive changes in the atomic structure of grain boundaries [58], healing of defects [58] and migration of divacancies and impurity atoms [59,60,61,62,63,64]. Examples of atom-number-conserving dynamical processes caused by an electron impact are shown in Figure 4. Although in all of these cases the frequency of the events and the fact that they can be controlled by placing the Ångström-size electron probe on the desired atom [61,64] clearly show that they are indeed driven by the electron irradiation, most of the observations have not yet been satisfactorily quantified through theory, possibly due to defect-related phonon modes or influence of ionization (or excitonic) events.

**Figure 5:**
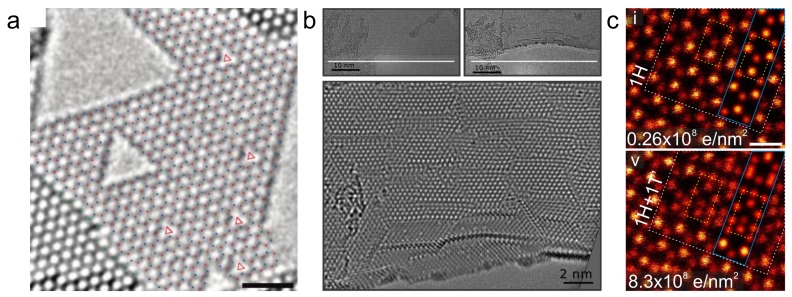
Electron-irradiation effects in non-graphene 2D materials. (**a**) Triangular vacancies formed into hexagonal boron nitride under electron irradiation at 80 kV. The red and blue marks stand for atoms of the two different elements. The scale bar is 1 nm. Reprinted with permission from Ref. [66], Copyright (2009) American Chemical Society. (**b**) Accumulation of defects close to an edge of MoS${}_{2}$ under 80 kV electron irradiation. In the last frame (bottom), the created vacancies have arranged into line-defects oriented with respect to the edge. Reprinted with permission from Ref. [68], Copyright (2013) American Physical Society. (**c**) Phase transition 1H→1T’ in MoTe${}_{2}$ caused by electron irradiation at 60 kV. Scale bar is 0.5 nm. From Ref. [70], https://pubs.acs.org/doi/10.1021/acs.chemmater.7b03760.

In contrast to graphene, in other 2D materials, bond rotation-type transformations are rare (but in some cases possible [65]), and the creation of vacancy-type defects is the most prominent structural change under electron irradiation. For example, in h-BN, electron irradiation leads to the formation of nitrogen-terminated triangular pores [52,66,67] (see Figure 5a). For transition metal dichalcogenides (TMDs), chalcogen vacancies are the most observed defects, often arranging into line defects [68] (see Figure 5b). A commonly observed structural change in TMDs is a local phase transition [69,70], typically between the 1H and 1T phases (see Figure 5c).

### 3.2. Low Energy Ions

**Figure 6:**
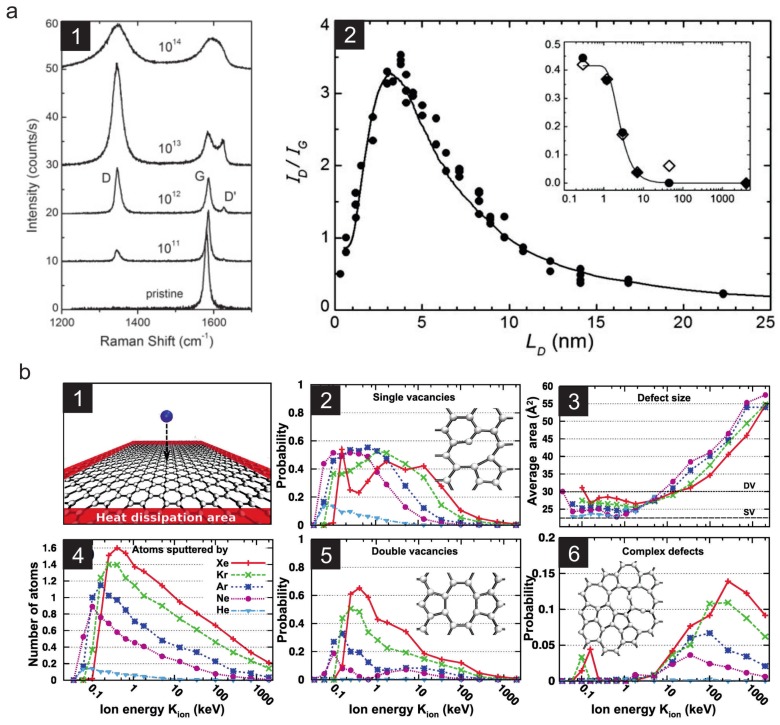
Defect creation in graphene by low energy ion irradiation. (**a1**) Evolution of the Raman spectra of graphene with increasing ion fluence in units of ions/cm${}^{2}$ (Ar${}^{+}$ with $E_{Kin}=90$ eV). (**a2**) The intensity ratio of the *D* and *G* peak $I_{D}/I_{G}$ as a function of fluence (here given in terms of the mean distance $L_{D}$ between two impacts) shows a clear maximum at the point where the structurally disordered regions begin to dominate. This behavior is in contrast to graphite, as shown in the inset in (**a2**). Reprinted from Ref. [71]. Copyright (2010), with permission from Elsevier. (**b**) Results of MD simulations of graphene evolution under noble gas ion irradiation. A significant probability to produce single (**b2**) or double vacancies (**b5**) in graphene is achieved at kinetic energies of (0.1–30) keV, where the nuclear stopping is at its maximum, compare with Figure 1a. More complex defects (**b6**) are rarely produced and only at higher energies. Reprinted with permission from Ref. [72], copyright (2010) by the American Physical Society.

As the first 2D material to be discovered, graphene was exposed to low energy ion beams soon after its discovery. In their seminal work [71] Lucchese et al. investigated how the Raman spectrum of irradiated graphene changes with increasing ion fluence, i.e., ions per unit area. The authors used 90 eV Ar${}^{+}$ ions which is with respect to defect creation one of the most efficient ion beams in the nuclear stopping regime as was shown by Lehtinen et al. [72], see Figure 6b.

The findings of Lucchese were not per se related to defect engineering, but established nevertheless an important tool for 2D material science. Their work demonstrated that the so-called Raman *D* peak, which in any sp${}^{2}$-coordinated graphitic system only occurs if otherwise symmetry-forbidden scattering processes are activated due to the presence of defects [73], clearly increases with increasing ion fluence up to a total fluence of ca. $10^{14}$ ions per cm${}^{2}$, see Figure 6a. Accordingly, with respect to the defect density the quality of any graphene sample may be conveniently assessed by Raman spectroscopy. Later on Eckman et al. tried to also characterize the type of defect by Raman spectroscopy [74] which however proved to be much more difficult.

According to the MD simulations [72,75], irradiation of graphene with noble gas ions up to energies in the range of some keV leads to the creation of single and double vacancies. Similar results have been obtained by MD simulations for carbon ions, where the probability to create single or divacancies remained constant at around 14% in the energy range from 10 to 100 keV projectile energy, and even lower below 10 keV. Larger openings like e.g., pores, can thus only effectively be created by accumulating defects in a spatially confined area by multiple ion impacts [76,77]. This effect has been successfully exploited e.g., for graphene patterning with high precision using the focused ion beam (FIB) from a helium ion microscope [75,78,79]. Note that also conventional FIBs based on Ga ions have successfully been used for patterning and functionalization of graphene [80,81] as well as for thinning of MoS${}_{2}$ [82], however the main drawback here is that with these ions typically unwanted collateral damage occurs. For example, in graphene edges are amorphized by the beam, probably due to the presence of residual gases [83] and tails of the focused ion beam.

At energies in the range of some keV, the ions pass through the 2D material so quickly that the momentum transfer from the ion to the target atoms is essentially symmetric over the plane, and hence occurs in the in-plane direction [72]. Under such conditions also the contribution of the chemical attraction between the ion and the target atoms becomes negligible and ion irradiation leads to the formation of higher order vacancies and amorphized areas, as was shown through focused ion beam processing of graphene at 35 keV using Ga${}^{+}$ ions [84]. Similar results were recently obtained by Yoon et al. [85] who studied ion irradiation of graphene with different noble gas ions and subsequent annealing through experiments and simulations.

**Figure 7:**
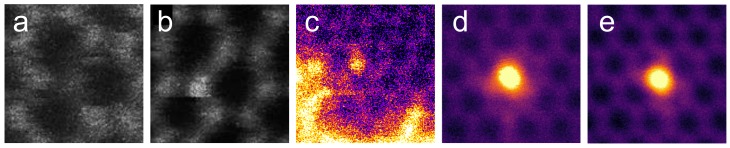
Ion-implanted impurity atoms in graphene, as imaged with scanning transmission electron microscopy. Due to the atomic-number dependent contrast, lighter atoms appear dark and heavier bright. (**a**) Boron (dark atom) implanted at 25 eV and (**b**) nitrogen (bright atom) implanted at 25 eV. Both images adapted with permission from Ref. [87], Copyright (2013) American Chemical Society. (**c**) Phosphorus (bright atom) implanted at 30 eV. The bright area at the bottom and on the left is caused by carbon-based contamination on graphene. Adapted from Ref. [92], licensed under a Creative Commons Attribution 3.0 License. (**d**,**e**) Three- and fourfold-coordinated implanted germanium atoms. Implantation energy was 20 eV. Reprinted with permission from Ref. [93]. Copyright (2018) American Chemical Society.

However, when reactive elements are used instead of noble gases at the lowest energy range (tens of eV), it is possible for the impinging ion to both displace a target atom from graphene and to be trapped in the created vacancy as an impurity atom. Examples for impurity atoms successfully implanted into graphene in this way are shown in Figure 7. This process was first predicted for boron and nitrogen through MD simulations in 2010 [86] and later demonstrated experimentally by Bangert et al. in 2013 [87]. Boron and nitrogen are natural dopants for carbon-based materials due to their similar size and one electron less and more, respectively. However, the most often observed impurity atom in graphene samples is silicon, which has been observed to both have a three-coordinated and a four-coordinated impurity configuration in the lattice [60,88,89,90]. It was predicted in 2015 [91], again through MD simulations, that it would also be possible to implant silicon atoms into graphene using low energy ion irradiation. However, to our knowledge, this has not yet been shown experimentally. Nevertheless, over the last years the number of implanted elements has been increased first by phosphorus [92] and later by germanium [93]. Bangert et al. also succeeded in implanting selenium into MoS${}_{2}$ using 10 eV ion irradiation [94]. In all reported studies, the major problem in ion implantation has been the simultaneously accumulated contamination, presumably adsorbed onto the sample due to momentum transfer from the ion beam to hydrocarbon molecules in the residual gas in the vacuum chamber. This complication has also made it difficult to establish accurate estimates for the ideal implantation energies for the various ions, but they appear to be close to the displacement threshold energy of graphene (ca. 21 eV [51]), which is also intuitively clear. Deviations from the displacement threshold value arise from the momentum transfer between the ion and the target atom, effects related to the chemical attraction between them, as well as the variation of the exact impact point of the ions with respect to the graphene unit cell.

Implanting foreign atoms is one way to manipulate the electronic and chemical properties of a given 2D material. Another way is the selective removal of atoms from the pristine material. This aspect is in particular important for the highly attractive class of 2D-TMDs. Due to the lower binding energy of the chalcogenide atoms compared to the transition metal atoms these are typically more easily removed from the 2D material upon irradiation. By combining MD simulations with DFT calculations it was shown for MoS${}_{2}$, that by choosing the correct irradiation parameters (ion type, energy, angle), one can even select from which layer the sulfur atoms will be removed [95]. The selective removal of sulfur atoms may not only be used to change the electronic properties from semi-conducting to conducting, it also gives rise to new electronic defect states. In the case of WS${}_{2}$ these have been claimed to be responsible for the experimentally observed linear increase in the near infrared adsorption of Ar${}^{+}$ ($E_{kin}=60$ keV) irradiated samples with increasing ion fluence in the range of ($10^{12}$–$10^{14}$) ions/cm${}^{2}$ [96].

Another important field of applications for defect-engineered 2D materials is the manufacturing of membranes for ultrafiltration purposes. The immense potential of porous graphene in this field was pointed out early on by several groups targeting innovative applications such as gas filtering [97,98], isotope separation [99,100], DNA sequencing [101], and water desalination [8]. This interest is driven by three advantages of graphene with respect to bulk materials. First, its ultimate thinness make it an ideal filtration membrane, because there is no friction to overcome. Any liquid or gas passing trough may be transported quasi-ballistically, drastically reducing power consumption in real applications. Second, the pore area necessary for improving state-of-the-art filtration techniques needs to be on the order of 1 nm${}^{2}$ or even smaller. This can easily be achieved in graphene by removing approximately ten atoms. Third, quantum mechanical effects do not only hinder even the lightest gases to pass through an intact graphene sheet, even a separation of ${}^{3}$He from ${}^{4}$He becomes feasible. As a consequence, there have been several experiments successfully conducted with graphene based membranes, many of which use energetic particles for the production of well-defined pores (see e.g., [102,103,104,105]). The general strategy of these approaches is to seal off a standard, commercially available mesoporous polymer membrane by a single layer of graphene. By subsequent irradiation with keV ions, defects are introduced into the graphene layer which then serves as the filtering element. This works quite well, and in fact much better than oxidative treatment or electron beam irradiation, but the number of graphene nanopores accidentally located on top of a mesoscopic pore in the supporting polymer film is quite low. While other pores also contribute to the filtration, they will not allow for ballistic transport. Also insufficient graphene coverage and intrinsic defect density pose challenges yet to overcome. According to MD simulations tailored nanopores in freestanding graphene may be created by low-energy ion irradiation under the condition that a few hundred ions hit the same spot which will however be difficult to achieve experimentally [76,77].

Interestingly, low energy ions can also be used to join two layers of graphene. Wu and coworkers demonstrated in their MD study that partially overlapping graphene layers under ion irradiation exhibit an increase of the tensile strength by a factor of two for fluences on the order of $10^{15}$ ions/cm${}^{2}$ carbon ions with $E_{Kin}=40$ eV [77,106]. The effect was attributed to an ion-induced cross-linking of the two layers by two different mechanisms: cross-linking due to coordination defects, i.e., interlayer C-C bonds, and, more importantly, due to ions trapped in between the layers. The latter effect is predicted to be enhanced for ions like carbon or silicon and to be absent for noble gas ions like Ar and He.

From the classical treatment of Rutherford scattering one can derive that the probability to transfer a given energy from the projectile to the target atoms decreases as the velocity of the projectile increases because the differential scattering cross section is inversely proportional to the square of the velocity, see Equation (3). Thus, by increasing the energy of the ions further, the defect creation efficiency due to nuclear stopping decreases, a counter-intuitive fact that is mirrored by the SRIM data shown in Figure 1a as well as the MD simulation data shown in Figure 6b. For electronic stopping to become dominant and effective, the energy of the projectile has to be much higher (see Section 3.3 below) which means that there is an intermediate regime where in fact both mechanisms may contribute to defect creation. In this regime the defect creation mechanisms are even less well understood as synergistic effects may occur. Despite the challenges to disentangle the relevant mechanisms and their respective contributions, there have been some experimental studies on ion irradiation in the medium energy range, e.g., the successful introduction of defects into graphene aerogels by 3.8 MeV He irradiation, which is interesting for energy applications [107] or the irradiation of supported graphene with 500 keV C ions [108]. The latter led to disorder of single layer graphene detectable by Raman and atomic force microscopy (AFM). The rather high fluences used in this study did not allow for analysis of individual defects, but it was found that the corrugation of single layer graphene first increases with increasing fluence (in contrast to the substrate) until it finally decreases again around $10^{14}$ ions/cm${}^{2}$ where graphene was found to adapt its shape to the substrate.

### 3.3. Swift Heavy Ions

Swift heavy ions are delivered by large scale accelerators the type of which determines the minimum and maximum achievable energy. Within this range the energy can in principle be chosen freely but often only a limited number of ion/energy combinations is available at a given accelerator. Furthermore, changing the energy requires a complete detuning of the accelerator which is often not possible within a given beam time. As an alternative, so-called degrader foils of a well-defined thickness can be used to vary the kinetic energy of the ions. However, this also changes the charge state and makes comparative experiments difficult. This is in particular true for 2D materials as the ions may not have sufficient time to achieve charge equilibration within the sample. Although all ion types are in principle available, in practice many accelerators operate with preferred ion species for which the corresponding sources run well. Available fluences and fluxes vary from accelerator to accelerator but usually allow for irradiations with (10${}^{8}$–10${}^{16}$) ions/cm${}^{2}$ within acceptable times. Often ion accelerators are not optimized for extremely low fluxes, making studies of individual ion impacts difficult. In addition, in most set-ups the beam is difficult to focus, although a few laboratories also operate dedicated set-ups, so-called microprobes, which may steer the beam with micrometer precision and even allow for single ion experiments [109,110].

**Figure 8:**
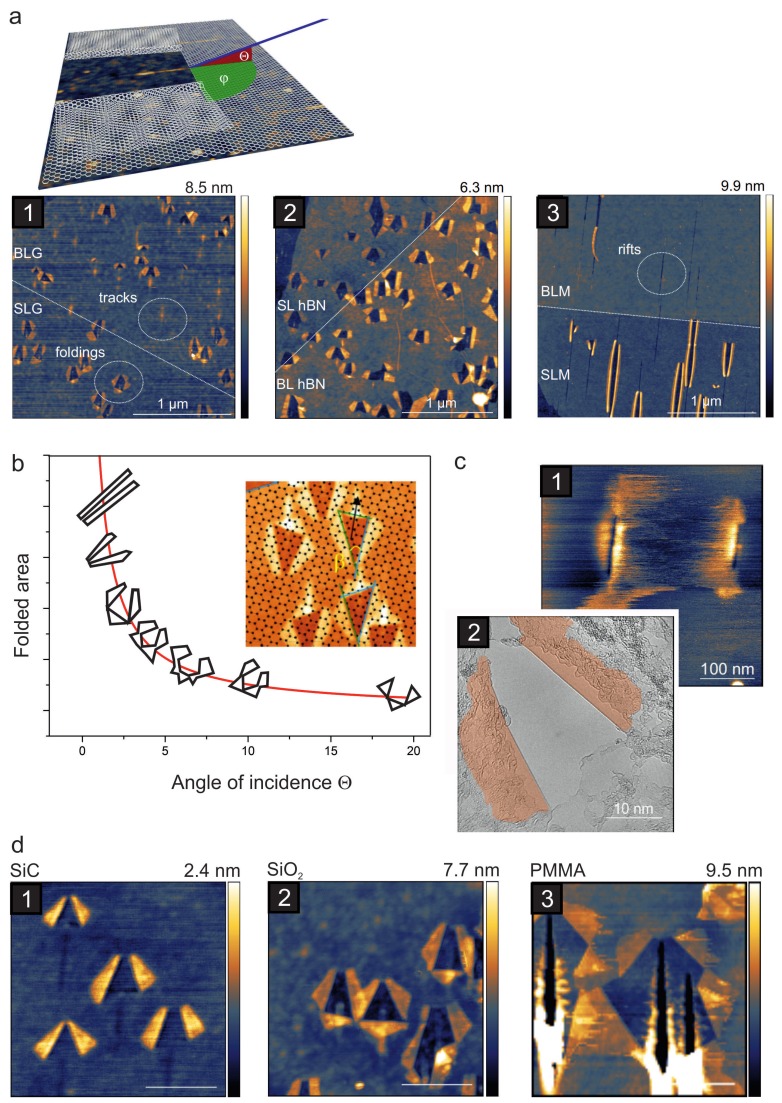
Folding of graphene and other 2D materials by SHI irradiation. (**a**) The sketch visualizes the backfolded graphene after an ion has impinged onto the surface under a grazing angle of incidence Θ. © IOP Publishing. Reproduced with permission from Ref. [112]. All rights reserved. Irradiation induced foldings as imaged by AFM in (**a1**) single and bilayer graphene, (**a2**) hBN, and (**a3**) MoS${}_{2}$, from Ref. [113]. (**b**) The shape of the graphene folding pattern (outlines shown in (**b**) are taken from AFM images) depends strongly on the angle of incidence. At larger angles the pattern consist of multiple foldings oriented along low-indexed crystallographic directions of the graphene [114] as shown in the inset in (**b**), while under very grazing incidence the azimuthal angle determines the direction of the two foldings, which are aligned along the ion trajectory, see Ref. [112]. (**c**,**d**): The substrate also influences shape and size of the foldings. AFM images of suspended graphene show slits (**c1**), which in fact are small foldings as can be seen in atomically resolved TEM images (**c2**); (**d1**) SiC-substrate, (**d2**) SiO${}_{2}$-substrate, (**d3**) Poly(methyl methacrylate)-substrate. Scale bars are 400 nm. © IOP Publishing. Reproduced with permission from Ref. [112]. All rights reserved.

The first experiments studying the effects of SHI irradiation on 2D materials were conducted with individual SHI impinging on graphene in a grazing incidence geometry. Akcöltekin et al. irradiated exfoliated graphene with 90 MeV Xe ions at an incidence angle of $\Theta =1^{\circ }$ with respect to the surface and investigated the samples by means of AFM [111]. They found unexpected and very characteristic morphological changes which turned out to be foldings of graphene. This is shown schematically in Figure 8a where foldings in graphene, hBN, and MoS${}_{2}$ are shown as well; note that a single ion has caused each of these massive structural changes involving hundreds of atoms.

In contrast, under perpendicular incidence no damage could be detected by ambient AFM. A more detailed investigation of this phenomenon showed that for graphene (i) the foldings occur up to a critical angle of $\Theta ≃20^{\circ }$; (ii) the size and shape of the foldings change with the angle of incidence and the larger the angle the more complex the folding pattern becomes; (iii) only at extremely oblique angles are the folding patterns exactly aligned along the ion beam; (iv) the probability for a folding to occur is unity for single layer graphene, but decreases drastically for bi- and tri-layers, and practically vanishes for thicker layers; (v) the substrate type strongly influences the folding (size, shape and probability). Figure 8 gives an overview of the most important results.

In view of defect engineering, the influence of the substrate is of particular interest and has been investigated by several groups via different means. The folding mechanism originally proposed by Alcöltekin included a direct defect creation in the graphene sheet generating a number of defects acting as a predetermined breaking point followed by the thermal expansion of the underlying substrate due to the electronic stopping. It was hypothesized that it is this thermal expansion of the substrate and probably also the interfacial water layer which pushes the graphene upwards. This was supported by the finding that in-situ prepared [115] as well as heated graphene samples with less interfacial water showed no tendency for foldings [116]. However, later Ochedowski et al. investigated suspended graphene irradiated with SHI by means of TEM and found that the foldings are an intrinsic response of the graphene itself in the sense that they do occur even without a substrate [112]. The foldings were much smaller – as was to be expected – but showed the characteristic morphology and alignment along the beam direction. In a theoretical study Zhao et al. modeled the SHI irradiation of supported graphene under grazing incidence by MD and found elongated openings in graphene, aligned along the ion trajectory. The study showed that the length of the openings increases with increasing angle of incidence in agreement with the experiments [117]. This data supports the assumption that substrate atoms add to the damage, but the study failed to reproduce the experimentally observed foldings.

While the folding seems to be an intrinsic response of graphene, it also happens in other 2D materials such as single layer MoS${}_{2}$ and hBN, both of which can be folded by grazing incidence SHI irradiation [116], see Figure 8a. Although this might be considered surprising due to the vastly different electronic properties that suggest a strong influence on the effective energy density, it becomes understandable when taking into account that all of these experiments were done on supported samples. While in the latter study no direct correlation between electronic properties and folding could be revealed, the mechanical properties seem to play an important role: Suspended and supported single layer graphene with an elastic bending modulus of 1.4 eV [118] folds easily and even (supported) tri-layers can still be folded. In contrast, the much stiffer MoS${}_{2}$ with an elastic bending modulus of 9.61 eV [118] cannot be folded if suspended. Also, supported MoS${}_{2}$ folds only if additional measures are taken: For single layer MoS${}_{2}$ to fold, the oblique SHI beam has to hit precisely along a low-indexed crystallographic direction. A folded MoS${}_{2}$ bilayer can only be obtained if it is pre-damaged by a previous ion impact [119].

The SHI-induced foldings represent a quite unique structure which could even be exploited in applications. For instance, they can be used as impact markers for fluence calibration purposes in materials that are otherwise insensitive to SHI irradiation or show only transient effects [120]. While the current understanding suggests that a substrate and favourable mechanical properties facilitate the folding process, the question why the folding occurs in the first place remains still to be answered.

**Figure 9:**
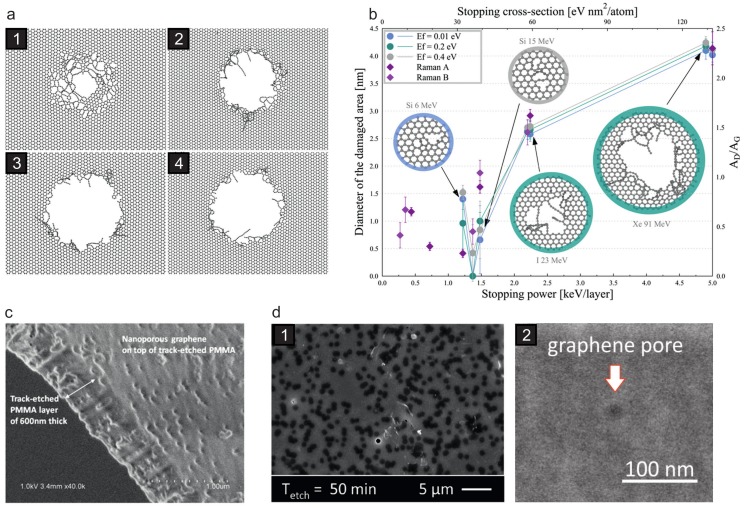
Pore creation in supported and suspended graphene by SHI irradiation. (**a**,**b**) MD simulations of SHI irradiation of graphene. (**a**) Results for graphene on a SiO${}_{2}$ substrate. Large pores can be created by individual impacts, the size of which can be controlled via the electronic stopping of the projectile. Data is shown for $dE_{el}/dx$ values of (**1**) 6.5 keV/nm, (**2**) 8 keV/nm, (**3**) 10 keV/nm, (**4**) 12 keV/nm. Only graphene is shown for clarity. Reprinted from from Ref. [117], Copyright (2015), with permission from Elsevier. (**b**) Results for graphene without substrate. Again, the pore size increases with increasing electronic stopping. Reprinted from Ref. [124], Copyright (2017), with permission from Elsevier. (**c**,**d**) Scanning electron microscope images from graphene/polymer membranes which have been irradiated with SHI and subsequently etched. Composite membranes with nanometer-sized pores in the graphene can be obtained in this way. (**c**) Reprinted from from Ref. [126], Copyright (2016), with permission from Elsevier; (**d**) Reproduced from Ref. [127] with permission from The Royal Society of Chemistry.

One of the major differences of SHI in comparison to low energy projectiles is the large amount of energy that may be deposited into an extended area. Graphene has proven to be quite resistant against damage caused by low energy projectiles (see Section 3.2 and below). Individual impacts of low energy ions create mostly point-like defects and suspended graphene has additionally a tendency to repair itself [121]. As carbon is an ubiquitous contamination, it is possible that low energy particle irradiation leaves no experimentally detectable damage. Thus, also for SHI irradiation one could on a first guess assume that graphene might be rather insensitive, because its high electric and thermal conductivity facilitate a fast spatial energy dissipation, yielding low effective energy densities. Zhao et al. studied supported graphene based on a combination of MD and continuum model calculations within the so-called two-temperature model (TTM) [122]. The TTM treats the energy transfer from the electronic system (subscript *e*) to the lattice (subscript *l*) via electron-phonon-coupling by a set of coupled differential equations, one for each subsystem:

(12) $$C_{e}\left(T_{e}\right)\frac{\partial T_{e}}{\partial t}\left(\vec{r},t\right)=\nabla \cdot \left[\kappa_{e}\left(T_{e}\right)\nabla T_{e}\left(\vec{r},t\right)\right]-g\left[T_{e}\left(\vec{r},t\right)-T_{l}\left(\vec{r},t\right)\right]+S\left(\vec{r},t\right)\;\mathrm{and}$$

(13) $$C_{l}\left(T_{l}\right)\frac{\partial T_{l}}{\partial t}\left(\vec{r},t\right)=\nabla \cdot \left[\kappa_{l}\left(T_{l}\right)\nabla T_{l}\left(\vec{r},t\right)\right]+g\left[T_{e}\left(\vec{r},t\right)-T_{l}\left(\vec{r},t\right)\right],$$

with electron-phonon coupling constant *g*, electronic and thermal conductivity $\kappa_{e,l}$ as well as electronic and thermal heat capacity $C_{e,l}$. They found that supported graphene sustains damage by individual SHI impacts above a threshold of $dE_{el}/dx=5$ keV/nm, although mainly due to pressure waves of the graphene/SiO${}_{2}$ system evolving from the impact volume, which again underlines the importance of the substrate. The defect structures in the graphene layer modeled with MD were nanopores, the size of which scales with the stopping power and is on the order of a nanometer (Figure 9a). For suspended graphene, the authors mention that it sustains extended damage for $dE_{el}/dx$ larger than a critical value of 8 keV/nm. According to this work, a few hundred carbon atoms can be easily removed per SHI, details of the resulting defect type and size were however not presented.

In general, modeling the interaction of SHI with a solid by MD is not an easy task, as it is not a priori clear how to treat the electronic excitation properly. In a first approach, one can simply distribute the energy corresponding to the electronic stopping among the atoms within a given radius (a parameter to be chosen) at the beginning of the MD simulation. The additional energy is instantaneously attributed to the atoms with randomly distributed momenta and then the equations of motion are integrated over a given time period. Very often, this results in a much too high energy density and the system explodes immediately. A more refined approach is to transfer the energy corresponding to the electronic stopping dynamically via the TTM into the lattice during the MD [123]. The coupling is accounted for in the MD when solving the equations of motion by an additional driving term.

This approach has been implemented and successfully used for 2D materials by Vazquez et al. in a TTM-MD simulation of SHI irradiation of suspended graphene under perpendicular incidence [124]. They showed that roundish pores are created in graphene, the size of which can be tuned by the electronic stopping power, i.e., the kinetic energy of the projectile. The threshold to create pores in graphene was determined to be around 3 keV/nm, see Figure 9b. Molecular dynamics simulations using the TTM are still hampered by the fact that the crucial parameters are usually ill-defined for any of the 2D materials and in addition depend on the temperature. For example, for the electronic thermal conductivity of graphene, values between 0.5 and 300 Wm${}^{-1}$K${}^{-1}$ have been reported, see Ref. [124], Refs. 70,71 therein, and [125].

These difficulties notwithstanding, the findings so far suggest that SHI irradiation is indeed a powerful defect-engineering tool suitable for pore creation in 2D materials. Direct experimental evidence, however, has turned out to be problematic. This is in part due to the fact, that no in-situ studies have yet been performed because suitable electron microscopes are usually not installed at the large scale accelerator facilities. Another problem still to be solved is the quality of the samples. For a detailed TEM investigation very minute areas of high quality graphene suffice, but for systematic irradiation studies much larger areas are needed. Furthermore, under perpendicular incidence roundish openings in the graphene sheet cannot be unambiguously distinguished from non-irradiation related defects due to, e.g., preparation and handling procedures. In fact, in the case of suspended graphene, so far only Raman data gives some evidence that indeed pores are created, the size of which changes with increasing stopping power [124].

A direct application of the defect engineering capabilities of SHI is easily found in the field of ultrafiltration as they offer a unique approach. Because of their extended range in solid matter (see Equation (6)), SHI can be used to drill a hole into graphene and the supporting polymer membrane all at once, i.e., in the same irradiation step. In contrast to other approaches based on low-energy ions (see Section 3.2) the polymer pores are widened only after the irradiation in a subsequent etching step which leaves the graphene pores unaffected. This concept has been recently successfully implemented [126,127], see Figure 9c,d.

Already the very first publication on graphene did include data from graphene based field effect transistors (FET) [7]. These devices show some unusual properties and have been investigated by many groups in great detail ever since. Studies concerned with ion irradiation of FET-devices based on 2D materials (2D-FET) represent a small but important field. On one hand, the transport characteristics of such devices are extremely sensitive to imperfections making them well-suited for sensing applications [128,129] but also for investigating defect-engineering techniques. While low-energy ion irradiation is typically detrimental for the device performance, at least two studies have shown that with SHI irradiation at not too high fluences, an improvement of the device in terms of mobility may occur, most likely because of a substrate-mediated annealing effect (see e.g., [130,131]). On the other hand, there is a particular interest in the radiation hardness of such devices to assess their applicability in radiation hard environments such as nuclear facilities or outer space. To study the latter, typically high-energy ions are used as these are present under the relevant conditions, where already individual hits may create severe damage, and the ions may pass protective layers rendering devices quickly inoperational. In addition, the defect-creation mechanisms are also comparable to those in neutron-radiation damage which is otherwise difficult to study.

The radiation hardness of 2D-FETs has been investigated experimentally. Ochedowski et al. exposed their devices to a 1 GeV uranium beam (the stopping power is almost at the maximum under these conditions, see green curve in Figure 1a), with fluences up to $4\times 10^{14}$ ions/cm${}^{2}$ and compared the effects on graphene-based and MoS${}_{2}$-based field effect transistors [132]. The former remained operational and even showed an improvement in conductivity at low fluences which was explained in terms of substitutional doping from the substrate and/or removal of adsorbates by the thermal spike. The latter showed no improvement and were rendered inoperational at the highest fluences. Also, inspection by AFM and Raman showed that SHI irradiation produced a much higher defect density than what one could achieve by keV ion irradiation. A similar experiment was later performed by Kim et al. who used a 10 MeV proton beam with comparable fluences [133]. As a consequence, the irradiated devices showed deteriorating performance with increasing fluence but remained operational up to the highest fluence of $10^{14}$ ions/cm${}^{2}$. Also, from Raman spectroscopy it was deduced that the MoS${}_{2}$ remained basically unchanged. The observed electrical changes were therefore not attributed to the MoS${}_{2}$ itself, but rather to the irradiation induced traps in the SiO${}_{2}$ substrate and at the SiO${}_{2}$/MoS${}_{2}$ interface.

Strictly speaking, a proton beam with a few MeV in kinetic energy is not the same as an SHI beam despite the high kinetic energy, as the electronic stopping power of protons is much smaller, see inset in Figure 1a. Nevertheless, electronic stopping still dominates over nuclear stopping for this type of projectiles as Mathew et al. could demonstrate in their fluence-dependent experiments with a 2 MeV proton beam (corresponding to $S_{e}$ = 32 eV/nm in graphite) on supported and suspended graphene samples of varying layer number [134,135]. The Raman data showed a significant difference in the damage of suspended and supported graphene, which was attributed to a reduced ion-induced electronically stimulated desorption in the case of suspended graphene. The threshold for damaging single layers of supported graphene was shown to be on the order of $10^{16}$ ions/cm${}^{2}$, which thus appears to be two orders of magnitude higher than for MoS${}_{2}$.

There have been only a few experiments with SHI of other 2D materials than graphene. Madauß et al. irradiated ultrathin MoS${}_{2}$ samples under grazing incidence [119]. The defect structure in bulk-like samples was found to be similar to what has been observed on other non-metallic bulk samples [136], i.e., chains of hillocks aligned along the incoming ion beam. In single and bilayer samples, however the defect pattern changed to either foldings (see Figure 8a3), area marked with "SLM") or rifts (see Figure 8a3), area marked with "BLM"), where material is obviously removed from the sample. These nano-incisions are also aligned along the incoming beam and showed a strong tendency for chemical reactions. More recently, the catalytic properties of such SHI irradiated Mo2${}_{2}$ single layers have been addressed in more detail. Madauss et al. analyzed the irradiated samples by means of AFM, SEM, X-ray photoelectron spectroscopy (XPS), and electrochemical measurements and complemented their study by theoretical simulations based on the TTM [137]. From the data, it was deduced that the irradiation with SHI not only produces nano-incisions with molybdenum-rich edges but that sulfur is removed from the basal planes as well due to the thermal spike in the underlying substrate. Qualitative analysis showed that per ion more than a thousand sulfur ions are removed and the corresponding samples show an enhanced catalytic activity with a low on-set value and high current densities.

### 3.4. Highly Charged Ions

Despite the versatility of HCI, only very few laboratories operate dedicated beamlines. The main drawback of HCI sources is the available ion flux. Sources based on electron cyclotron resonance can deliver higher currents but are limited with respect to the obtainable maximum charge state. Sources based on an electron beam ion trap can deliver very high charge states, up to naked uranium, but often with very small currents of a few pA or even less. This may be one of the reasons, why there are not too many studies of 2D materials irradiated with highly charged ions yet. The first experiment was conducted by Hopster et al. who irradiated exfoliated MoS${}_{2}$ with HCI [138]. The analysis proved to be difficult because of the chosen substrate (KBr), which is by itself rather sensitive to HCI irradiation [139]. The analysis by AFM showed both pits and protrusion induced by individual ion impacts. The pits where created in the KBr substrate while the protrusions showing up in the AFM images were shown to be a combination of a topographic hillock and an area of enhanced friction. A more systematic AFM study was later conducted by Hopster et al. who irradiated graphene exfoliated on SiO${}_{2}$. The authors showed that similar to earlier studies of HCI irradiated graphite [140], defects in graphene do not show up in the topography but only in the so-called friction force mode. In this mode also chemically modified areas may be detected [141]. It was shown that the defect radius increases with increasing potential energy of the ions, that a minimum potential energy was needed to create a detectable defect, and also indications for a decrease in size with increasing kinetic energy were found. The authors hypothesized that the interaction time was the determining factor for the latter.

**Figure 10:**
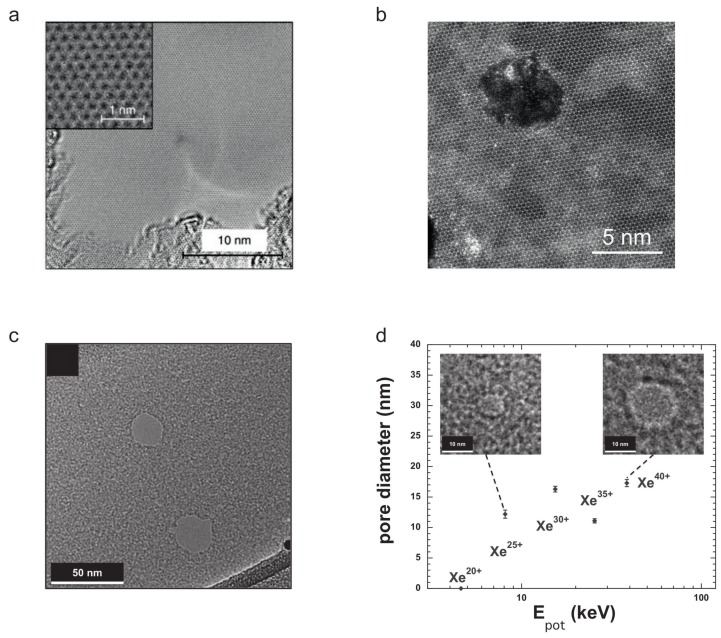
Examples for effects of HCI irradiation of 2D materials. (**a**) Graphene irradiated with 180 keV Xe${}^{40+}$ ions at a fluence of $10^{12}$/cm${}^{2}$ remains largely unaffected. From Ref. [41], licensed under a Creative Commons Attribution 4.0 International License. (**b**) In contrast, MoS${}_{2}$ irradiated also with 180 keV Xe${}^{40+}$ exhibits round nanometer-sized pores. From Ref. [144]. (**c**,**d**) Irradiation of ultrathin carbon nanomembranes with HCI. The HCI can be used to create pores of various sizes in the membrane depending on the chosen charge state, i.e., the potential energy of the projectile. Reprinted from Ref. [147], with the permission of AIP Publishing.

Charge exchange processes of HCI in graphene have been shown to be extremely fast. Gruber et al. measured the exit charge state of HCI passing through a suspended graphene layer and as a result almost all projectiles were found to be neutral [41]. From the data analysis, it was deduced that the projectile captures and stabilizes tens of electrons within less than a few fs and that graphene has to sustain current densities of more than $10^{12}$ A cm${}^{-2}$ to deliver a sufficient number of electrons at such short time scales. The graphene samples were analyzed by HRTEM afterwards and surprisingly, no signs of defects could be found in the irradiated samples, see Figure 10a. This obvious lack of defects after irradiation stands in clear contrast to data from supported samples. For example, graphene-FETs irradiated with HCI show a significant change in their transport characteristics [142]. Starting at fluences as low as 15 ions per μm${}^{2}$ the electron and hole mobilities decrease. The reduction of mobility scales with the potential energy of the ions and the carrier density increases with the fluence. A similar result was obtained by Peng et al. who investigated HCI-irradiated graphene by Raman spectroscopy [143]. These data show that indeed HCI can be used for defect engineering and that the difference to suspended graphene is most likely due either to substrate-induced damage, or to the difficulties in tracing defects, and/or graphene’s efficient self-repair mechanisms.

The question about the exact nature of HCI-induced defects in graphene remains an open one. In terms of theory, it appears even less clear how to describe the energy transfer from the electronic system to the lattice correctly. However, simply considering the energy density, creation of extended defects such as pores seems feasible. For example, a Xe${}^{40+}$ ion deposits 38.5 keV of potential energy within a volume with a depth of a few nanometers. This is clearly above the threshold of 3 keV/nm, established for SHI damage of graphene, even if we take a reduced efficiency into account. However, at least in graphene there is no clear indication so far, that extended defects are created by HCI irradiation. In fact, most experimental evidence points at a defect type which is connected to a change from sp${}^{2}$- to sp${}^{3}$-bound carbon. This may very well be due to the unique thermal and electronic properties of graphene and therefore other 2D materials appear better suited to demonstrate the defect-engineering potential of HCI.

A natural choice would be hBN, the graphene-analogue with respect to structure. First experiments investigating the effect of HCI irradiation of single-layer hBN show similar results as for graphene: HCI-induced defects show up as nanometer-sized areas of enhanced friction, but are otherwise not resolved in ambient AFM. Kozubek at al. showed by secondary ion mass spectrometry (SIMS) that boron atoms are removed from the lattice by HCI irradiation and that their yield increases drastically with increasing charge state [144,145]. The sublimation of boron could be successfully modeled using the TTM. For this study, the source term in Equation (12) was adapted to the particular case of a HCI introducing potential energy into the impact site:

(14) $$S\left(\vec{r},t\right)=bE_{pot}G\left(t\right)F\left(\vec{r}\right),$$

where G(t) is a Gaussian function with a width of 1 fs describing the temporal energy distribution. The spatial distribution of the energy $F(\vec{r},t)$ is given by a half sphere, the radius of which is treated as a fitting parameter. The parameter *b* is used to scale the energy density.

Another example for successful material manipulation by HCI are ultrathin carbon nanomembranes (CNM) which are not electrically conducting [146] and thus should be more prone to damage than graphene. Ritter et al. irradiated CNMs with HCI of varying charge states and found nanometer-sized round openings, the size of which scaled with the potential energy of the HCI [147], see Figure 10c,d. These pores have relatively sharp edges and a small size distribution making them attractive for filtration purposes. Another example for successful pore creation in 2D materials via HCI irradiation is single layers of TMDs. Irradiation of suspended single layer MoS${}_{2}$ samples with HCI leads to pores of similar size and shape [144] while in bilayers of WS${}_{2}$ triangular shaped pores with atomically sharp edges have been observed. These types of defects are not easily produced by any other particle beam and underlines again the unique possibilities HCI offer in terms of defect engineering. Systematic studies of HCI-induced defects in 2D materials and the corresponding creation mechanisms are thus very promising and will certainly be conducted in the near future.

## 4. Open Problems

Defect engineering by particle irradiation is a well-established and effective technique in material science that has been proven to be applicable in the field of 2D materials with equal success, though the number of 2D systems studied along these lines remains limited. While in defect-engineering strategies for 3D materials one may often neglect the surface and thus the ambient conditions, this is not an option with 2D materials. This brings along the requirement for extremely clean and very high quality suspended samples in order to truly exploit their full potential. To avoid artefacts from contamination, experiments should ideally be carried out in-situ under ultra high vacuum, which requires the operation of the appropriate methods within the same set-up, e.g., an ion source combined with a TEM or a Raman set-up. Note, that even then ubiquitous carbon may cover up defects, constitute a source for carbon atoms to heal defects or even prevent defect creation in the first place. Therefore, well-defined preparation and cleaning protocols for 2D materials need to be established for novel defect-engineering strategies to be reliable and effective. For the more conventional 2D materials, i.e., graphene and TMDs, this has already been achieved and implemented with some success. However, these procedures need to be tailored and optimized for any given material. Because the number of 2D materials available to researchers steadily increases [148] (see, e.g., recent work on two-dimensional MXenes [149], natural minerals [150], and ores [151]), the development and implementation of appropriate procedures remains an open challenge for the future.

The theoretical description of defect-creation mechanisms in 2D materials by elastic electron scattering and keV ion irradiation is overall well developed, allows to put forward predictive statements, and the agreement with experimental data is quite good. This is however definitely not the case for the more unconventional ion beams, i.e., SHI, HCI, and ions in the intermediate regime where synergistic effects of electronic and nuclear stopping may occur. There have been some efforts lately to model the effects of SHI and HCI ions on 2D materials as discussed above. However important issues remain to be solved, e.g., how to treat the charge of the projectile correctly or how to choose the correct area (or in the case of multiple-layer samples the correct volume) of energy deposition. Similarly, excitonic and ionization-related effects of inelastic electron scattering on the atomic structure of 2D materials are currently poorly described with the existing methods. Further developments on these topics would help to strengthen the use of these methods and thus to extend the scope of defect-engineering strategies for 2D materials.
